# Identification of Urban Leprosy Clusters

**DOI:** 10.1155/2013/219143

**Published:** 2013-10-30

**Authors:** José Antonio Armani Paschoal, Vania Del'Arco Paschoal, Susilene Maria Tonelli Nardi, Patrícia Sammarco Rosa, Manuela Gallo y Sanches Ismael, Eduvaldo Paulo Sichieri

**Affiliations:** ^1^Department of Architecture and Urbanism, University of São Paulo, Avenida Trabalhador São-Carlense, 400, Arnold Schimidt, 13566-590 São Carlos, SP, Brazil; ^2^Nursing Department, School of Medicine of São José do Rio Preto-FAMERP, Avenida Brigadeiro Faria Lima 5416, Vila São Pedro, 15090-000 São José do Rio Preto, SP, Brazil; ^3^Institute Adolfo Lutz, Regional Laboratory, Rua Alberto Sufredini Bertoni 2325, Maceno, 15060-020 São José do Rio Preto, SP, Brazil; ^4^Lauro de Souza Lima Institute, Rod Comandante João Ribeiro de Barros, km 225, 17034-971 Bauru, SP, Brazil; ^5^Mercator Engineering and Consulting GIS, Rua Voluntários de São Paulo 3439, 15015-200 São José do Rio Preto, SP, Brazil

## Abstract

Overpopulation of urban areas results from constant migrations that cause disordered urban growth, constituting clusters defined as sets of people or activities concentrated in relatively small physical spaces that often involve precarious conditions. *Aim*. Using residential grouping, the aim was to identify possible clusters of individuals in São José do Rio Preto, Sao Paulo, Brazil, who have or have had leprosy. *Methods*. A population-based, descriptive, ecological study using the MapInfo and CrimeStat techniques, geoprocessing, and space-time analysis evaluated the location of 425 people treated for leprosy between 1998 and 2010. Clusters were defined as concentrations of at least 8 people with leprosy; a distance of up to 300 meters between residences was adopted. Additionally, the year of starting treatment and the clinical forms of the disease were analyzed. *Results*. Ninety-eight (23.1%) of 425 geocoded cases were located within one of ten clusters identified in this study, and 129 cases (30.3%) were in the region of a second-order cluster, an area considered of high risk for the disease. *Conclusion*. This study identified ten clusters of leprosy cases in the city and identified an area of high risk for the appearance of new cases of the disease.

## 1. Introduction

Urbanization of the population and the growth of city outskirts constitute the dominant demographic scenario, making control of intraurban transmission of some endemic diseases even more complex [1]. The use of geotechnology and the availability of digital maps of cities is a breakthrough for the integrated planning of different sectors [2, 3]. Studies on the spatial distribution of diseases, particularly in respect to the urban pattern, are gradually being performed in Brazil [4–6].

The investigation of clusters, a set of people or activities concentrated in a relatively small physical space, may help our understanding of circumstances of focal problems [5, 7]. Studies of spatial clusters and the ecological model, based on the idea of interrelating factors, if perfected, may be a promising alternative to expand our understanding of the transmissibility of disease and even improve resource allocation in healthcare [8]. As leprosy, a public health problem, is still an endemic disease that is neglected, knowledge of its spatial distribution and an understanding of clusters, particularly in municipalities that have controlled the disease for years, may contribute to improve epidemiological surveillance measures in a given location [7].

The city chosen for this study is located in the northwest of the state of São Paulo, Brazil. It has a population of 408,258 inhabitants and a total area of 431.30 km^2^; 119.48 km^2^ is urbanized with 100% of the streets surfaced with tarmac. A total of 99% of the households have running water and 95% have sewage collection. The entire area has regular collection and transportation of solid waste (garbage) from both residential and commercial properties. The city has a well distributed and diverse healthcare service with eight hospitals and a total of 1749 hospital beds, 1612 doctors, and 29 government healthcare clinics. Demographic and social indicators of the municipal are comparable with those of developed countries. The city has a human development index of 0.834 which is considered high by the United Nations Development Program [9].

The incidence of leprosy in the city in 2011 was 5.1/100,000 with 21 new cases diagnosed and a prevalence of 4.7/100,000 [10]. Although studies show that leprosy, though endemic, is declining in Brazil, an investigation by Penna et al. [7] using the geoprocessing of new cases shows that there are outbreaks of recent transmission especially in the nine states that make up the Amazon region. The result of this study triggered a series of interventions by the federal government in this region including financial, technical, and scientific measures.

When data of a town divulges a low prevalence of a specific disease, city planners generally reduce allocated resources and do not continue with control measures. Moreover, health professionals and the general population forget the basic characteristics of the disease, such as the signs and symptoms, and thus, diagnosis is delayed which can facilitate transmission [11].

Hence, the aim of this study was to investigate the presence of clusters by means of the residential grouping of people who have or have had leprosy.

## 2. Methods

This was a population-based, descriptive, cross-sectional, and ecological study. Four hundred and twenty-five addresses of residents in the municipality from a total of 478 leprosy cases diagnosed in the period from January 1, 1998 to December 30, 2010 and treated as part of the Leprosy Control Program were geocoded after their addresses had been confirmed by home visits.

Fifty-three patients were excluded from the study as their addresses were incomplete, nonexistent, or in rural areas or because the patient was not a resident in the municipality.

The home visit was exclusively to confirm the address given by the patient. It is important to mention that the municipality is a reference center for 101 neighboring towns; thus, some people from other regions use the address of relatives or acquaintances to attain treatment. In recent years, this issue has been solved with the checking of national databases such as the National System for Notifiable Diseases (SINAN) which prevents patients from other regions from being included as residents in the city.

### 2.1. Geocodification of Data

*X* and *Y* coordinates were allocated to confirmed addresses. This process, developed using the MapInfo software, was created from the interpolation of the coordinates of a street compared with the address of the patient.

 With the longitude and latitude being known, it was possible to export data to another system employed to analyze cases and group those with similar features. The concepts of Burrough [12] were used; the cartographic base is the spatialization of geographical features (point, line, or polygon) with attributes, for example, a street name that has coordinates (*X*, *Y*) in a given map projection system [12].

### 2.2. Spatial Analysis and Statistics of Geographic Data

 The software CrimeStat, which works with point samples of geocoded data, was used to determine the presence of clusters [13]. The first input parameters are the geographical location of the addresses of leprosy cases, that is, the latitude and longitude.

Two other very important parameters used in the study were the clinical classification of leprosy and year of onset of treatment entered as weight and time, respectively. The weight and the time are values associated with the *X* and *Y* positions of each point. If we consider that each patient has a different clinical classification and year of treatment, it is possible to correlate these variables to the geographical location of each patient's address and group cases by similar characteristics. Thus, each mapped point receives a differentiated statistical treatment. Thus, in the same cluster there may be patients for whom the risk of transmission of the disease was high and the proximity between them was highly significant, but the year of starting treatment was different.

The use of these parameters allowed the search for first-order clusters to be refined; at least eight points were needed to define a cluster [14]. The CrimeStat software (version 1.1) employs two procedures to analyze point patterns: the distance to the nearest neighbor and the K-function [13]. These two methods analyze the properties of data, such as “second-order clusters” or spatial dependency (represented in the figure as light gray).

Cluster analysis was performed employing the K-function by estimating the kernel intensity with a maximum separation of 300 meters between cases (represented as dark grey ellipses proportional to 1.5 standard deviations) [13, 15]. The distance between cases was delineated by the authors of this paper as it appears that no published studies have defined this distance previously. The clinical forms of the disease were classified using the criteria of Ridley and Jopling [16].

After consent and approval of the Research Ethics Committee of the Medical School in São José do Rio Preto, data were extracted from the Hansen/FAMERP/CNPq project database [17] complemented with data from 2007 to 2010.

## 3. Results

Maps prepared using geoprocessing tools, spatial analysis techniques, and statistical methods showed the distribution of 425 leprosy cases between 1998 and 2010 stratified by the clinical classification of the disease and the year of starting treatment.

**Figure 1:**
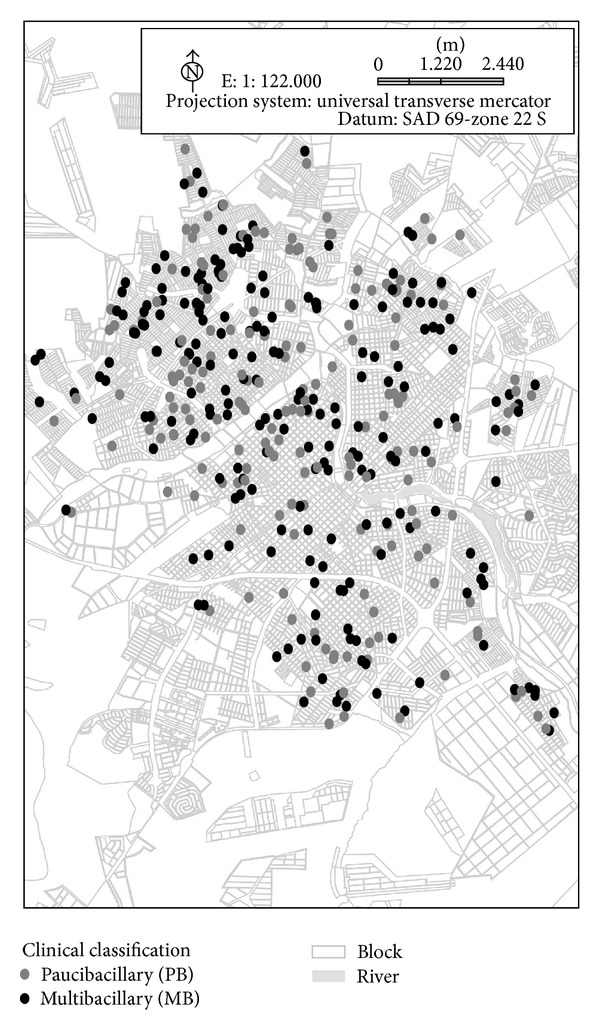
Spatial distribution of leprosy cases paucibacillary (PB) and multibacillary (MB) treated between 1998 and 2010 according to disease transmission criteria.

The distribution of cases is throughout the entire city; Figure 1 shows the location of residences of patients over time (from 1998 to 2010) for both communicable forms (borderline borderline leprosy, borderline lepromatous leprosy and clinical forms, and lepromatous leprosy; *n* = 228) and noncommunicable forms (intermediate leprosy, tuberculoid leprosy, and borderline tuberculoid leprosy; *n* = 197).

### 3.1. Formation of Clusters

Ten clusters of residences of leprosy patients were identified with a gradual increase in the number of cases within the clusters between 1998 and 2010. The dark-grey ellipse-shaped figures (proportional to 1.5 standard deviations) represent clusters of at least eight cases with up to 300 meters between residencies, and the large light-gray region, the second-order cluster, is the location of highest risk.

The spread of the locations of clusters identified by the K-function shows that there are several groups of points with very similar characteristics both in value (clinical classification) and in time (year of starting treatment).

**Figure 2:**
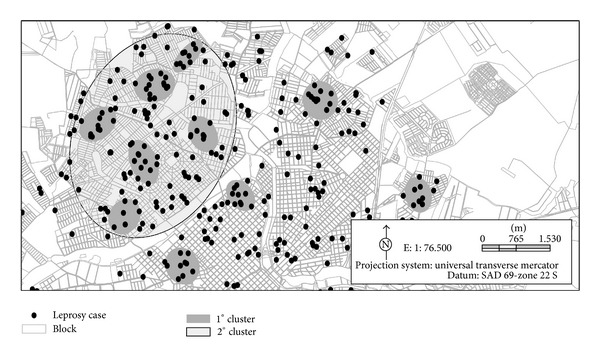
Constitution of the ten clusters formed by grouping the residences of leprosy cases as first order (dark gray) and second order (light grey) in the period from 1998 to 2010 in São José do Rio Preto, SP.

The second-order cluster, identified in light gray, specifies a new “cluster” that brings together smaller groups with very peculiar proximities and characteristics (Figure 2).

In this study, a large cluster (second order) was identified in the northern region of the city indicating that the pattern of the leprosy cases identified in the first-order clusters is not random; there is spatial dependency in the studied variables.

Ninety-eight (23.1%) of the 425 geocoded cases were located within one of the 10 clusters that were identified in this study, and 129 (30.3%) were in the region covered by the second-order cluster; this is an area considered of high risk for the disease. The population of the region covered by the second-order cluster was estimated to be 129,230 inhabitants in 2009 [9].

Among the first-order clusters, four leprosy cases lived in the same residence as other cases and in the second-order cluster; three cases lived at the same address. All the cases were native to the region.

Table 1 shows the evolution of leprosy cases according to the year of starting treatment and residing in the identified clusters (first- and second-order).

The neighborhoods where the largest volume of clusters was identified, as well as the largest number of patients per cluster, were urban areas with the highest population density, that is, in the north and northeast of the city with 227 (53.4%) of the patients living in the area of highest risk, thus, maintaining the profile of this region.

It was also observed that there were a constant number of cases in the areas identified as clusters (between 32.0% and 80.6%) over the 13 years of this study (Table 1).

## 4. Discussion

The geoprocessing technique showed a gradual but uneven increase in new cases in the city and identified the formation of ten first-order clusters, and a large area was considered of high risk for the disease consisting of groups of leprosy cases.

The methods used for spatial and statistical analysis of the geographic data to determine the presence of groups of cases clearly identified clusters. The method considers the premise that “things that are close are more similar than things at a distance and therefore belong to the same group.” This theory, in addition to assessing location and quantity, also correlates values of mapped points using statistical validation methods, and subsequently, it places some specific points in groups [14]. The search for trends and the identification of clusters of leprosy cases are useful complements to other traditional disease control measures.

Of the 425 cases existing in the municipality, 227 (53.4%) were found in the 10 clusters identified in this study. The spatial distribution of leprosy cases in the municipality over these years, according to the clinical form of the disease, helps our understanding of the transmission of the disease and provides a basis for further studies on this issue. The distribution of the clinical forms and the areas covered were verified, thereby, improving the time needed to start monitoring cases, the logistics of medication distribution, and the development of health education measures.

The tools used in this study support the development of more specific strategies and consequently provide resource optimization and also an improvement in the understanding of factors associated with the occurrence of the disease chiefly in large urban centers where the concentration of cases is high.

The city of this study is one of the best cities in the country; it is the 10th richest city in the country and 3rd in the state in terms of quality of life. With life expectancy at 71.3 years, the city has an excellent infrastructure and an income per capita of R$ 512.01 (US$ 248.26) [9] (Secretaria Municipal 2011). Even so, there are social inequality and vulnerable populations resulting in the presence of clusters as identified in this study. Higher rates of leprosy cases in areas with precarious socioeconomic conditions have been reported by other studies [18, 19].

In this study, the interrelationship or intersectoral approach to strengthen the technical capacity [20] has created an interface between health and engineering, which, when carrying out the visits, raised important issues and factors that may have contributed to the contagion in the domiciliary setting or long-stay environments [21].

Other studies should be conducted to determine and prevent forms of contagion that may occur within long-stay environments, in particular, within the household, the work place, and on public transport used every day by the same groups of people possibly resulting in long periods in contact with leprosy carriers.

This study shows that the distribution of leprosy cases in recent years has reached the whole municipality; however, the greatest concentration of cases, where the largest volume of clusters was identified, is located in the northern and northeastern regions of the city where there are the greatest socioeconomic difficulties [9].

The residences located in nonlegalized urban areas were one of the greatest problems in data collection and analysis in this study as the methodology used addresses and official figures. Due to the lack of accurate data, some patients were excluded from the sample, even though strategies to solve problems related to illegal housing areas can be designed within geotechnology. This is important as the “excluded” population also has difficulty to access public healthcare services, and in an analysis of this magnitude, these individuals remain excluded from public health.

In several clusters, situations were identified where cases were neighbors with their homes contiguously linked (either back to back or side to side) often with entrances in different streets and without the residents ever having had any type of relationship or even knowledge of each other's existence and definitely without knowledge of the disease or the form of contagion [22].

One of the causes of the strong tendency of infection of this disease may be related to the precarious quality of life demonstrated by the overpopulation of poorer neighborhoods, exacerbated occupation not only of the urban infrastructure but also, in particular, of the occupation of areas not officially allocated for urbanization.

When urban indexes exceed those predicted by calculations of civil engineering, architecture, and urbanism, there is a lack of all social amenities in the area, including public health, social assistance, schools, play schools, and community facilities. In many situations, people are obliged to move to other neighborhoods for the basic infrastructure. Thus, they are exposed to all kinds of human clustering.

All related aspects, especially the overpopulation of regions, whether planned urban neighborhoods or unofficial housing areas, constitute elements that can be regarded as ingredients of a series of conditions that increase the likelihood of the spread of leprosy. And thus, the unsanitary conditions and subhuman *modus vivendi* may be one factor that contributes to the emergence of this disease [22].

The identification of the formation of urban clusters of leprosy determined by territorial space and characterized by clinical form together with the time at onset of the disease seems to be innovative in research. The same innovation occurred in other studies that used geotechnology as an important tool to demonstrate findings. Sakamuri et al. [23] demonstrated the geographic distribution and genetic frequency of *Mycobacterium leprae* using polymerase chain reaction (PCR). Moreover, Duarte-Cunha et al. [24] analyzed the spatial pattern of the occurrence of leprosy in relation to the decentralization of treatment centers and campaign strategies, and Cury et al. [19] evaluated the relationship between the presence of leprosy cases and low socioeconomic conditions rather than population density.

The discussion of the fragility of health information in some Brazilian states is related to two fundamental points. Initially, it is expected that inequalities in the reporting of vital and healthcare events are an indicator of the organization of epidemiological surveillance systems and are not subordinate to the computer tools used. In fact, at a local level, it is possible to have an adequate surveillance system even without the digitalization of data. However, the systematic collection and reporting of vital and healthcare events are indispensable tools for decision-making in relation to the definition of healthcare policy, obtaining public funds for the sector, and monitoring the effect of diseases on health, in the evaluation of the impact of prevention measures and the quality of services provided [25].

## 5. Conclusion

The statistical methods employed identified ten clusters of leprosy cases in the city and identified a possible area of high risk for the appearance of new cases of the disease. The different aspects highlighted by the results of this study demonstrate the need for further research and active pursuit of cases in the municipal, in particular in the areas identified by clusters. Discussions with political and administrative authorities of the city should be intensified in order to stimulate scientific research and provide preventive interventions to control leprosy and other diseases in the population.

## Figures and Tables

**Table 1:** Distribution of new leprosy cases in the clusters from 1998 to 2010, according to the new cases geoprocessed and the estimated values of population/year.

Year of starting treatment	New cases of leprosy (Geoprocessed 1998–2010)	New cases of leprosy residents in clusters	%	Estimated population in the municipal (inhabitants)^a^
1998	30	17	57.7	343,059
1999	44	28	63.6	351,944
2000	36	17	47.2	357,862
2001	43	30	69.8	367,247
2002	45	25	55.6	374,745
2003	41	15	36.6	382,274
2004	41	19	46.3	398,079
2005	32	23	71.9	400,826
2006	25	8	32.0	415,508
2007	25	15	60.0	402,770
2008	22	13	59.1	414,272
2009	13	5	38.5	419,632
2010	25	12	48.0	408,258

Mean	32	17	53.1	—
Median	32	17	—	
Total	**425**	**227**	**53.4**	**—**

^a^Source: city office of São Jose do Rio Preto, http://www.riopreto.sp.gov.br/.
